# Quantitative NanoLC/NSI^+^-HRMS Method for 1,3-Butadiene Induced *bis*-N7-guanine DNA-DNA Cross-Links in Urine

**DOI:** 10.3390/toxics9100247

**Published:** 2021-10-02

**Authors:** Luke Erber, Samantha Goodman, Caitlin C. Jokipii Krueger, Ivan Rusyn, Natalia Tretyakova

**Affiliations:** 1 Department of Medicinal Chemistry and Masonic Cancer Center, University of Minnesota, Minneapolis, MN 55455, USA; lerber@umn.edu (L.E.); purox003@umn.edu (C.C.J.K.); 2 Department of Veterinary Integrative Biosciences, College of Veterinary Medicine and Biomedical Sciences, Texas A&M University, College Station, TX 77843, USA; sgoodman@cvm.tamu.edu

**Keywords:** urinary DNA adducts, 1,3-butadiene, *bis*-N7G-BD, LC-MS/MS, mercapturic acids

## Abstract

1,3-Butadiene (BD) is a common environmental and industrial chemical widely used in plastic and rubber manufacturing and also present in cigarette smoke and automobile exhaust. BD is classified as a known human carcinogen based on evidence of carcinogenicity in laboratory animals treated with BD by inhalation and epidemiological studies revealing an increased risk of leukemia and lymphohematopoietic cancers in workers occupationally exposed to BD. Upon exposure via inhalation, BD is bioactivated to several toxic epoxides including 3,4-epoxy-1-butene (EB), 3,4-epoxy-1,2-butanediol (EBD), and 1,2,3,4-diepoxybutane (DEB); these are conjugated with glutathione and excreted as 2-(N-acetyl-L-cystein-S-yl)-1-hydroxybut-3-ene/1-(N-acetyl-L-cystein-S-yl)-2-hydroxybut-3-ene (MHBMA), 4-(N-acetyl-L-cystein-S-yl)-1,2-dihydroxybutane (DHBMA), and 1,4-*bis*-(N-acetyl-L-cystein-S-yl)butane-2,3-diol (*bis*-BDMA). Exposure to DEB generates monoalkylated DNA adducts, DNA-DNA crosslinks, and DNA-protein crosslinks, which can cause base substitutions, genomic rearrangements, and large genomic deletions. In this study, we developed a quantitative nanoLC/NSI^+^-HRMS methodology for 1,4-*bis*-(gua-7-yl)-2,3-butanediol (*bis*-N7G-BD) adducts in urine (LOD: 0.1 fmol/mL urine, LOQ: 1.0 fmol/mL urine). This novel method was used to quantify *bis*-N7G-BD in urine of mice treated with 590 ± 150 ppm BD for 2 weeks (6 h/day, 5 days/week). *Bis*-N7G-BD was detected in urine of male and female BD-exposed mice (574.6 ± 206.0 and 571.1 ± 163.4 pg/mg of creatinine, respectively). In addition, major urinary metabolites of BD, *bis*-BDMA, MHBMA and DHBMA, were measured in the same samples. Urinary *bis*-N7G-BD adduct levels correlated with DEB-derived metabolite *bis*-BDMA (*r* = 0.80, Pearson correlation), but not with the EB-derived DNA adducts (EB-GII) or EB-derived metabolites MHBMA and DHBMA (*r* = 0.24, *r* = 0.14, *r* = 0.18, respectively, Pearson correlations). Urinary *bis*-N7G-BD could be employed as a novel non-invasive biomarker of exposure to BD and bioactivation to its most mutagenic metabolite, DEB. This method will be useful for future studies of 1,3-butadiene exposure and metabolism.

## 1. Introduction

1,3-Butadiene (BD) is a common environmental and industrial chemical widely used in plastic and rubber manufacturing [1] that is also present in cigarette smoke [2] and automobile exhaust [3]. BD is classified as a known human carcinogen based on the evidence of tumorigenicity in laboratory animals, mechanistic studies revealing genotoxic effects, and epidemiological data [4]. Studies with experimental animals revealed that inhalation exposure to BD induces tumors in multiple organs including heart, liver, lung, stomach and kidney in mice [5] and pancreas, thyroid, kidney, mammary gland, uterus and testes in rats [6]. Workers occupationally exposed to 1,3-butadiene and polymer industries are at an increased risk of leukemia and lymphohematopoietic cancers [7,8,9,10].

**Scheme 1:**
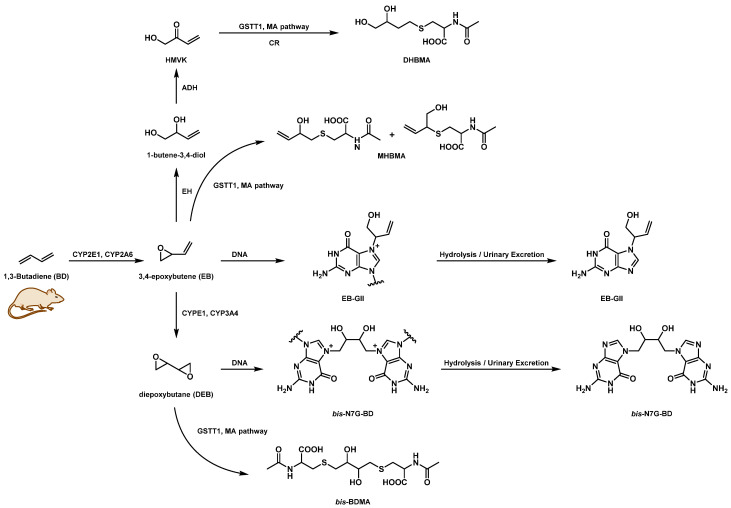
Partial metabolic scheme of 1,3-butadiene showing DNA adduct formation, hydrolysis and urinary excretion. ^a^CR, carbonyl reductase; EH, epoxide hydrolase; EB-GII, N7-(1-hydroxy-3-buten-2-yl) guanine; MA, mercapturic acid; bis-N7G-BD, 1,4-*bis*-(gua-7-yl)-2,3-butanediol; bis-BDMA, 1,4-*bis*-(N-acetyl-L-cystein-S-yl)butane-2,3-diol; MHBMA, 2-(N-acetyl-L-cystein-S-yl)-1-hydroxybut-3-ene/1-(N-acetyl-L-cystein-S-yl)-2-hydroxybut-3-ene; DHBMA 4-(N-acetyl-L-cystein-S-yl)-1,2-dihydroxybutane.

BD undergoes metabolic activation to a number of DNA reactive epoxides, including 3,4-epoxy-1-butene (EB), 1,2,3,4-diepoxybutane (DEB), and 3,4-epoxy-1,2-butanediol (EBD) (Scheme 1) [11,12]. These reactions are catalyzed by cytochrome P450 monooxygenases CYP2E1 and CYP2A6. Previous studies have revealed metabolic deactivation pathways of BD-derived epoxides. EB and EB-derived hydroxymethylvinyl ketone (HMVK) are conjugated to glutathione, processed via the mercapturic acid pathway, and are ultimately excreted in urine as 2-(N-acetyl-L-cystein-S-yl)-1-hydroxybut-3-ene/1-(N-acetyl-L-cystein-S-yl)-2-hydroxybut-3-ene (MHBMA) and 4-(N-acetyl-L-cystein-S-yl)-1,2-dihydroxybutane (DHBMA), respectively (Scheme 1) [13]. DEB can be inactivated through hydrolysis by epoxide hydrolase (EPHX1) or conjugation with glutathione (GSH) [14,15,16]. In the latter pathway, DEB is conjugated to two glutathione molecules and processed via the mercapturic acid pathway, ultimately excreted in urine as 1,4-*bis*-(N-acetyl-L-cystein-S-yl)butane-2,3-diol (*bis*-BDMA) (Scheme 1) [17].

If not detoxified, EB, DEB, and EBD alkylate DNA bases to form a range of nucleotide adducts which can interfere with accurate DNA replication [18,19]. Of all BD-derived epoxides, DEB has the greatest genotoxic potential in vitro and in vivo. Compared to monoepoxide metabolites, DEB is nearly 200-fold more mutagenic, likely due to its bifunctional properties [7]. DEB can enter cellular nuclei to react with guanine nucleotides in DNA and form N7-(2-hydroxy-3,4-epoxybut-1-yl)-guanine monoadducts. The latter can be hydrolyzed to N7-(2,3,4-trihydroxybut-1-yl)-guanine (N7-THBG) or can alkylate neighboring nucleotides to form bifunctional DNA adducts such as DNA-DNA and DNA-protein crosslinks (Scheme 1) [20,21]. These crosslinked adducts can block DNA replication and cause base substitutions, chromosome rearrangements, and large genomic deletions [22,23].

Previous mouse BD inhalation studies quantified the most abundant DEB-specific bifunctional DNA adduct, 1,4-*bis*-(gua-7-yl)-2,3-butanediol (*bis*-N7G-BD) (3.95 ± 0.89 adducts/10^7^ nucleotides (nts)); at a lower abundance are N7G-N1A-BD (0.27 ± 0.07 adducts/10^7^ nts) and 1,*N^6^*-HMHP-dA (0.044 ± 0.008 adducts/10^7^nts) [24,25]. *Bis*-N7G-BD cross-links are hydrolytically labile and are spontaneously released from DNA via depurination (t_1/2,_ 81.5 h) [25,26].

Due to the potent carcinogenicity and mutagenicity of BD and the widespread exposure of humans to this toxic chemical, sensitive biomarkers of BD exposure and bioactivation to DEB in vivo are needed. Previous studies by Georgieva et al. [27,28] characterized a number of DEB-specific biomarkers, specifically the N-terminal valine hemoglobin (Hb) adduct, *N,N*-[2,3-dihydroxy-1,4-butyl]valine (*pyr*-Val). Pyr-Val adducts were observed in blood of F344 rats and B6C3F1 mice exposed to low levels of BD (0.5 ppm) (mice: 8.0–17.4 pmol/g globin, rats: 0.6–0.9 pmol/g globin) [28] and in blood of occupationally exposed workers (0.08–0.86 pmol/g globin) [29]. Because protein adducts are not repaired, Hb-based biomarkers represent cumulative exposure to DEB over time (red blood cell lifetime is approximately: 45 days in mice, 63 days in rats, and 120 days in humans) [30].

Urinary mercapturic acids have been commonly used as sensitive, non-invasive biomarkers of carcinogen exposure and as a quantitative measure of their bioactivation to DNA-reactive intermediates [31]. BD-mercapturic acids (*bis*-BDMA, MHBMA, DHBMA) have been measured in urine of rats, confirmed smokers, and workers exposed to BD occupationally and reported as biomarkers of exposure to BD [17,32,33,34,35]. Furthermore, these markers permitted evaluation of BD-derived electrophilic metabolite formation in vivo [17].

Even though urinary metabolites and hemoglobin adducts of BD are sensitive biomarkers, they provide information on the exposure and not necessarily on the toxicologically relevant internal dose of DEB. By contrast, DNA adducts are mechanistically relevant, cancer-related markers; DNA is the ultimate biological target of genotoxic carcinogens. We have previously reported analytical methods for sensitive detection of many butadiene-DNA adducts in genomic DNA, [24,25,36,37,38] however, large epidemiological studies do not make DNA available. In the present work, we have developed a quantitative nanoLC/NSI^+^-HRMS methodology for *bis*-N7G-BD DNA adducts in urine. We reason that urinary *bis*-N7G-BD can be used as a biomarker of exposure to BD and its bioactivation to the genotoxic metabolite DEB. Urinary DNA adducts were chosen because unlike tissue or blood samples, urine can be collected in a non-invasive manner, easily stored and transported, and is readily available from epidemiological and animal studies. Our quantitative method for *bis*-N7G-BD utilizes a high resolution capability of an Orbitrap mass spectrometer for accurate, specific and sensitive quantitation of the *bis*-N7G-BD. Following method validation, the new method was applied to quantify *bis*-N7G-BD in urine of mice exposed to BD by inhalation.

## 2. Experimental Section

### 2.1. Materials

HPLC grade methanol and LC-MS grade water, acetonitrile and methanol were purchased from Fisher Scientific (Pittsburgh, PA, USA). Synthetic urine was obtained from Dyna-Tek Industries (Surine 720, Dyna-Tek Industries, Lenexa, KS, USA). Common solvents and chemicals were purchased from Sigma-Aldrich (St. Louis, MO, USA). Isolute ENV+ reversed phase SPE cartridges (40 mg/1 mL) were purchased from Biotage (Charlotte, NC, USA). Oasis HLB cartridges were purchased from Waters (Milford, MA, USA). *bis*-N7G-BD and ^15^N_6_-*bis*-N7G-BD standards were synthesized as previously described [26,38,39]. Molar concentrations of *bis*-N7G-BD and ^15^N_6_-*bis*-N7G-BD in standard solutions were measured using UV spectrophotometry (Cary 3500 UV-Vis Multicell Peltier, Agilent Technologies, Santa Clara, CA, USA) according to the published molar extinction coefficient (ε) of 15,700 M^−1^ cm^−1^ at 252 nm at pH 1 [26]. Isotopic purity of ^15^N_6_-*bis*-N7G-BD as determined by mass spectrometry (Orbitrap QExactive, Thermo Scientific, Santa Clara, CA, USA) was 99.82%. Stock solutions of *bis*-N7G-BD and ^15^N_6_-*bis*-N7G-BD were prepared in water and stored at −20 °C. MHBMA, DHBMA, D_6_-MHBMA, and D_7_-DHBMA were purchased from Toronto Research Chemicals (Toronto, Ontario, Canada). Bis-BDMA and D_6_-bis-BDMA were synthesized in our laboratory as previously described [17].

### 2.2. Animal Study

Ten female and ten male adult mice (a total of 20 animals between 8–12 weeks old upon arrival) of the Collaborative Cross (CC) mouse strain CC079 were obtained from the University of North Carolina Systems Genetics Core (Chapel Hill, NC, USA). Mouse strain CC079 was previously used in a study of inter-strain variability upon exposure to 1,3-butadiene in CC mice [40]. Male and female mice were housed separately under identical conditions. At start of exposure male and female mice weighed 27.6 ± 4.1 g and 19.8 ± 3.6 g respectively. Mice were housed with their littermates (2–4 per cage). Each cage was kept in a temperature-controlled room (24 °C) and approximately 50% humidity at the Texas A&M Institute for Genomic Medicine (College Station, TX, USA) animal facility. Cages were exposed to a 12-h light/dark cycle. Mice were fed with standard rodent chow (Global 2019 Extruded (irradiated format) rodent diet, Teklad Diets, Madison, WI, USA) with purified water ad libitum. Prior to the experiments, all animals were cleared to be pathogen-free and acclimated to the facility for 30–45 days. Male and female mice (5 per group) were exposed to either filtered air or 590 ± 150 ppm BD for 2 weeks (6 h/day, 5 days/week) using whole-body exposure chambers as reported previously [41]. Exposures were conducted separately for female and male mice on consecutive 2-week periods. All experimental procedures involving animals and their husbandry were approved by the Institutional Animal Care and Use Committee (IACUC) of Texas A&M University. Urine collection was performed using metabolic chambers (32 × 25 × 36.5 cm; Techniplast USA, West Chester, PA, USA). Urine was collected on the morning of day 3 and day 10 of the two-week exposure, transferred to microcentrifuge tubes and stored at −80 °C until analysis. Creatinine levels in each urine sample were quantified using the Parameter^TM^ Creatinine Assay Kit (R&D Systems, Minneapolis, MN) according to the manufacturer instructions.

### 2.3. Sample Processing and bis-N7G-BD Adduct Enrichment

*Bis*-N7G-BD adducts were purified from mouse urine using solid phase extraction (SPE) followed by offline HPLC. Urine (10 μL) was first centrifuged at 10,000 g for 15 min to remove particulate matter. The urine samples were spiked with ^15^N_6_-*bis*-N7G-BD (10.0 fmol, internal standard) in 100 μL of water. SPE-based purification of *bis*-N7G-BD was accomplished using Isolute ENV+ cartridges (40 mg/1 mL; Biotage). The SPE columns were first conditioned with 2 mL methanol and 2 mL Milli-Q water. Urine samples (110 μL) were loaded onto the conditioned columns. Next, the columns were sequentially washed with 2 mL water, 1 mL 0.1% formic acid in water, and 1 mL 3% acetonitrile in water. Both *bis*-N7G-BD and ^15^N_6_-*bis*-N7G-BD were eluted with 1 mL 60% methanol in water before drying under nitrogen.

SPE-enriched *bis*-N7G-BD and ^15^N_6_-bis-N7G-BD were further purified using offline HPLC. HPLC blanks were injected after every three samples to monitor analyte carryover. Offline HPLC was performed using the Agilent 1100 series HPLC system containing the Zorbax Eclipse XDB-C18 column (4.6 × 150 mm, 5 μm), a UV detector and automated fraction collector (Agilent Technologies). SPE-enriched samples were first resuspended in 100 μL of water containing 2.1 nmol 2′-deoxythymidine (dT) and 2.1 nmol deoxyadenine (dA) as HPLC retention time markers. The HPLC flow rate was set at 0.9 mL/min using 0.4% formic acid in Milli-Q water (A) and 0.4% formic acid in HPLC-grade acetonitrile (B). To accomplish online HPLC separation, the solvent gradient was started at 0% B for the first 8 min and then linearly increased to 16% B in 9 min and then to 50% B in 3 min. Solvent B was then reduced to 0% B in 5 min before equilibrating at 0% B for an additional 5 min for a total separation time of 30 min. The retention time markers dT and dA were monitored using UV absorbance at 254 nm. Using this method, dA eluted at 14.6 min, *bis*-N7G-BD eluted at 15.5 min, dT eluted at 16.6 min. HPLC fractions containing *bis*-N7G-BD and ^15^N_6_-*bis*-N7G-BD (15.2−15.9 min, 0.7 mL) were collected into 1.7 mL Eppendorf tubes (Thermo Fischer Scientific, Waltham, MA, USA). HPLC fractions were dried under vacuum and resuspended in 0.01% acetic acid (16 μL).

### 2.4. NanoLC/NSI^+^-HRMS Analysis of Urinary bis-N7G-BD Adducts

NanoLC/NSI^+^-HRMS quantitative analysis of *bis-*N7G-BD was performed using a nano-LC HPLC instrument (Dionex UltiMate 3000 RSLCnano HPLC system, Thermo Fisher Scientific) coupled with a Orbitrap QExactive mass spectrometer instrument (Thermo Scientific, Santa Clara, CA, USA). The nanoLC instrument was equipped with a picofrit nano-column (0.075 × 200 mm; New Objective, Littleton, MA, USA) self-packed with Zorbax C18 beads (5 μm; Agilent Technologies). Solvent A was 0.01% acetic acid in LC-MS grade water and solvent B was a LC-MS grade mix (1:1) of acetonitrile and methanol. Samples were resuspended in 0.01% acetic acid in LC-MS grade water (16 μL). The sample injection volume was set at 4 μL and blank samples were injected after every 3 samples to monitor analyte carryover. For analyte separation, the solvent gradient started at 2% for 7.5 min at a flow rate of 0.8 μL/min. At 7.5 min, the flow rate was reduced to 0.3 μL/min in 0.5 min. Then, the gradient was linearly increased to 25% B in 9 min and then 50% B in 10 min. Next, the solvent composition was reduced to 2% B in 3 min. Finally, the flow rate was increased to 0.8 μL /min in 1 min and held at 2% B for 6 min for column equilibration for a total separation time of 37 min.

Quantitative analyses were achieved in the positive ion mode using parallel reaction monitoring (PRM) mode by fragmenting [M + H]^+^ ions of *bis*-N7G-BD in the high collision dissociation (HCD) cell using the normalized collision energy (NCE) of 30 and an isolation width of 1.0 amu. The mass spectrometry source conditions were optimized with infusion of authentic *bis*-N7G-BD to maximize instrument sensitivity. The resulting fragment ions were detected in the mass range of *m/z* 100–420 using the Orbitrap mass analyzer (HRMS) at a resolution of 70,000 following an AGC target of 2E5 or a maximum injection time of 50 ms. Quantitative analyses of *bis*-N7G-BD were conducted in the parallel reaction mode using fragment ions corresponding to the neutral loss of guanine from protonated molecules of *bis*-N7G-BD (*m/z* 389.1426 [M + H]^+^→238.0934 [M + H − Gua]^+^), and the formation of protonated guanine (*m/z* 389.1426 [M + H]^+^→152.0567 [Gua + H]^+^). The ^15^N_6_-labeled internal standard was analyzed analogously using the transitions *m/z* 395.1248 [^15^N_6_-M + H]^+^→241.0844 [^15^N_6_-M + H − ^15^N_3_-Gua]^+^ and 155.0477 [^15^N_3_-Gua + H]^+^. Quantitation was based on the peak areas obtained from extracted ion chromatograms. The ratio of the analyte peak area divided by the internal standard peak area was multiplied by the amount of internal standard spiked into the urine sample and expressed as pg/mL urine. *Bis*-N7G-BD amounts was calculated using (A_AN_/A_IS_ x 10.0 fmol spiked internal standard), where A_AN_ represents the analyte peak area and A_IS_ represents the internal standard peak area. Adduct levels were normalized to the creatinine and expressed as μg/mg creatinine.

### 2.5. Method Validation

**Figure 1:**
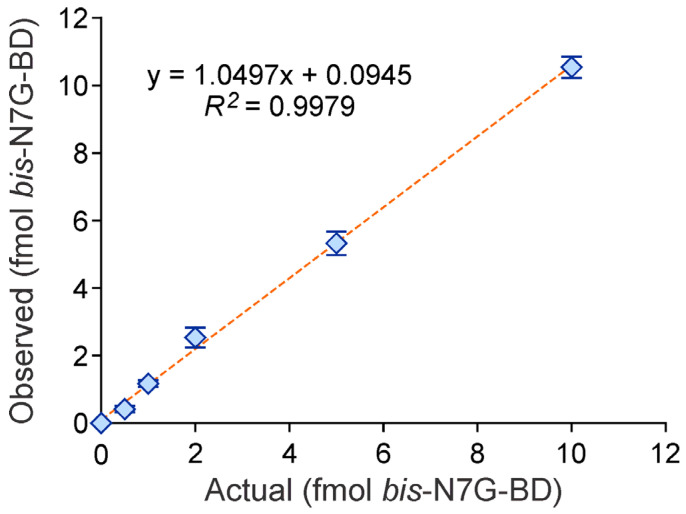
NanoLC-NSI^+^-HRMS method validation: correlation analysis between the spiked and the observed amounts of *bis*-B7G-BD spiked into control mouse urine (10 μL). Spiked amounts were 0, 0.5, 1.0, 2.0, 5.0, or 10.0 fmol of *bis*-N7G-BD and 10.0 fmol of ^15^N_6_- *bis*-N7G-BD (internal standard), followed by sample processing and nanoLC-NSI^+^-HRMS analysis.

For method validation, standard curves were generated by spiking control mouse urine or synthetic urine (10 μL) with a fixed amount of ^15^N_6_-*bis*-N7G-BD (10.0 fmol) and increasing amounts of *bis*-N7G-BD (0.1–10.0 fmol, in triplicate). Standard curves were generated upon nanoLC/NSI^+^-HRMS analysis of samples processed via SPE and offline HPLC. The observed levels of *bis*-N7G-BD amounts were plotted against the theoretical values (Figure 1). Standard curves were processed by regression analyses.

Method limit of detection (LOD) and limit of quantitation (LOQ) were determined by spiking control urine from unexposed mice (10 μL) with *bis*-N7G-BD (0.1–10.0 fmol) and ^15^N_6_-*bis*-N7G-BD (10.0 fmol) in triplicate. Spiked urine samples were processed and analyzed as described above. Method LOD was calculated as the level of *bis*-N7G-BD resulting in a signal to noise (S/N) ratio > 3, and the method LOQ was assigned to the amount of *bis*-N7G-BD resulting in a signal to noise (S/N) ratio > 10 and a percent coefficient of variation (% CV) less than 15%.

Intra- and inter-day precision and accuracy of the method were identified by spiking control mouse urine (10 μL) with either 1.0 fmol or 5.0 fmol of *bis*-N7G-BD and 10.0 fmol of ^15^N_6_-*bis*-N7G-BD internal standard. The samples were analyzed in triplicate on three consecutive days. The intra- and inter-day precision value was calculated from the relative standard deviations between these measurements. Method accuracy was calculated using the formula (A_m_/A_a_ x 100%), where A_m_ represents the measured amount of *bis*-N7G-BD and A_a_ represents the actual amount of analyte spiked.

Analyte recovery from SPE and offline HPLC cleanup steps was quantified in the following approach. Control mouse urine (10 μL, in triplicate) was spiked with ^15^N_6_-*bis*-N7G-BD internal standard (10.0 fmol). Unlabeled *bis*-N7G-BD was added before or immediately after SPE purification, and after by sample cleanup by offline HPLC. These recovery samples were processed by the nanoLC/NSI^+^-HRMS analysis method described above. Analyte recovery was calculated by comparing the ^15^N_6_-*bis*-N7G-BD:*bis*-N7G-BD peak area ratios.

The analyte signal suppression in the urine matrix was quantified using the nanoLC/NSI^+^-HRMS analysis method described above. Analyte signal suppression was calculated by comparing the ratio of peak area of *bis*-N7G-BD (10.0 fmol) spiked into the control non-exposed mouse urine matrix following sample processing and the corresponding peak area of *bis*-N7G-BD pure standard spiked in pure water.

To test the stability of bis-N7G-BD in mouse urine, a freeze–thaw stability assay was performed as previously described for 1,3-butadiene urinary DNA adducts [36]. Urine from 1,3-butadiene exposed mouse was frozen (−20 °C) for 24 h and thawed at room temperature for 2 h (1 freeze–thaw cycle). This sample was processed and analyzed in triplicate. Two additional freeze–thaw cycles were performed, each sample was followed by processing and analysis in triplicate. Variability in freeze–thaw cycles was calculated using %CV.

### 2.6. Quantitation of bis-BDMA

Mouse urine samples (5 μL) were mixed with 50 mM ammonium formate buffer (pH 2.5, 100 μL) and formic acid (10 μL). 6 μL of 10 ng/μL solution of D_6_-*bis*-BDMA internal standard in water was added, and the samples were centrifuged at 13,000 rpm. for 15 min. The supernatant was loaded onto Isolute ENV+ cartridges (1 mL, 50 mg) and processed as previously described [17]. SPE eluates were dried with a speedvac and redissolved in 30 μL of water. 4 μL of this solution was injected onto the HPLC column for HPLC-ESI^−^-MS/MS analysis.

HPLC-ESI^−^-MS/MS analysis of *bis*-BDMA was achieved using a Dionex UltiMate 3000 RSLC nanoHPLC system (Thermo Fisher Scientific) coupled with a Thermo-Finnigan TSQ Quantum Discovery mass spectrometer (Thermo Fisher Scientific). HPLC separations and mass spectrometry source conditions were set up as previously described [17]. Mass spectrometry data were acquired using the selected reaction monitoring mode (SRM). The SRM transitions employed were *m/z* 411.1→282.1, 411.1→153.1 and 411.1→128.1 (*bis*-BDMA) and *m/z* 417.1→285.1, 417.1→153.1 and 417.1→131.1 (D_6_-*bis*-BDMA). HPLC-ESI^−^-MS/MS methodology was fully validated by analyzing control mouse urine spiked with known amounts of *bis*-BDMA and D_6_-*bis*-BDMA. Adduct levels were normalized to the creatinine measurements.

### 2.7. Quantitation of MHBMA and DHBMA

Mouse urine samples (20 μL) were vortexed and mixed with 200 μL of milliQ water, 20 μL 1 N HCl and spiked with 6 μL of 10 ng/μL solutions of D6-MHBMA and D7-DHBMA standards in water. The samples were centrifuged at 13,000 rpm for 15 min and processed by SPE on Waters Oasis HLB cartridges as previously described [14]. The SPE eluates were dried, redissolved in 30 μL of 0.1% formic acid, and processed by HPLC-ESI^−^—HRMS/MS using a Pursuit 3 Diphenyl column (2.1 × 150 mm, 3 µm) eluted with a gradient of 0.1% formic acid and acetonitrile as previously described [14].

Quantitative analysis of BD-mercapturic acids was conducted on a QExactive Orbitrap interfaced with a Dionex 3000 nano LC system (Thermo Scientific) in the negative ion mode using parallel reaction monitoring (PRM) mode by fragmenting [M−H]^−^ ions of MHBMA and DHBMA in the high collision dissociation (HCD) cell using the normalized collision energy (NCE) of 25 and 35, respectively, and an isolation width of 1.0 amu. The mass spectrometry source conditions were optimized with infusion of authentic standards to maximize instrument sensitivity. The resulting fragment ions were detected in the mass range of *m/z* 50–400 using the Orbitrap mass analyzer (HRMS) at a resolution of 70,000 following an AGC target of 2E5 or a maximum injection time of 100 ms. MHBMA was detected using a scan event consisting of fragmentation of *m/z* 232.1 to *m/z* 103.0223. D6-MHBMA internal standard was detected using a scan event consisting of fragmentation of *m/z* 238.1→109.0600. DHBMA was detected by fragmentation of *m/z* 250.1→121.0329. D7-DHBMA were detected through the scan events consisting of fragmentation of *m/z* 257.1194→128.0768. HPLC-ESI^−^-HRMS methodology was validated by analyzing control mouse urine spiked with known amounts of MHBMA, D6-MHBMA, DHBMA, and D7-DHBMA. Adduct levels were normalized to the creatinine measurements.

### 2.8. Statistical Analyses

Statistical analyses of data was completed using GraphPad Prism (v 9.0 San Diego, CA, USA). Results are presented as mean +/− standard deviation compared for urinary *bis*-N7G-BD, *bis*-BDMA, MHBMA, and DHBMA levels. The significance threshold was set at *p* < 0.05 for all tests performed. Pearson’s partial correlations (r) were reported for correlation studies between urinary *bis*-N7G-BD, N7-(1-hydroxy-3-buten-2-yl) guanine (EB-GII) levels and concentrations of BD-mercapturic acids.

## 3. Results

### 3.1. NanoLC/NSI^+^-HRMS Method Development for bis-N7G-BD in Mouse Urine

**Scheme 2:**
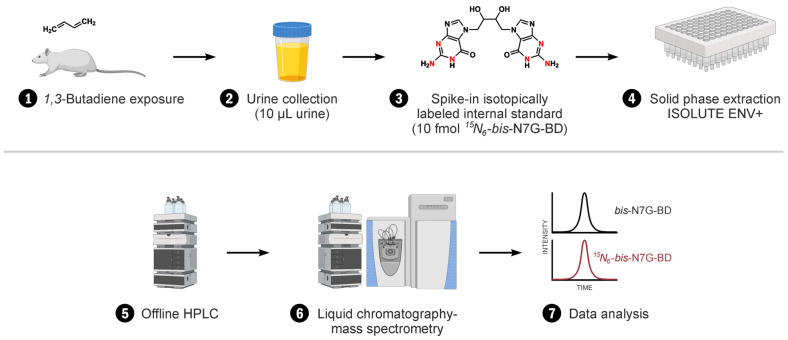
Experimental scheme for isotope dilution nanoLC-NSI^+^-HRMS analysis of *bis*-N7G-BD in urine.

Urine is a complex biological matrix containing many metabolites and metabolic byproducts [42]. Urine contains bile salts, polar molecules and other small molecules that can adversely impact NSI analyses via signal suppression [43,44]. Therefore, trace analyses of urinary nucleotide adducts require analyte-specific purification strategies such as SPE and offline HPLC fractionation [45,46]. In this study, we adopted the following experimental approach to quantify bis-N7G-BD in mouse urine (Scheme 2). Urine samples are spiked with ^15^N_6_-*bis*-N7G-BD internal standard and then processed by Isolute ENV+ SPE columns to remove the bulk of impurities. Next, samples are enriched using offline HPLC prior to quantitative analysis by nanoLC/NSI^+^-HRMS. Spiking samples with isotopically labeled internal standard at the start of the analysis accounts for any analyte losses during sample processing and permits accurate analyte quantitation.

For the first step of *bis*-N7G-BD purification from the urine matrix, several SPE cartridges were tested. These cartridges included Strata X (Phenomenex), Oasis HLB (Waters) and Isolute ENV+ (Biotage). Of these SPE cartridges, Isolute ENV+ was effective with respect to eliminating co-eluting impurities and analyte recovery. Isolute ENV+ SPE recovery for *bis*-N7G-BD was calculated as 83.0 ± 4.7% in spiked control mouse urine samples.

Our previous experience with developing methods to quantify urinary DNA adducts showed that SPE alone does not result in sufficient sample purity for nanoLC/MS analyses [36]. Therefore, following SPE, samples were further purified using an offline HPLC method [39]. This method was modified by adding formic acid to buffer B (0.4% formic acid in ACN) to eliminate the pH gradient in the original method. The gradient program was modified to be 0% B to 16% B in 9 min. Third, the total run time was reduced from 40 min to 30 min. This updated method permitted separation of *bis*-N7G-BD from the EB-derived DNA adduct (EB-GII), which is much more abundant than *bis*-N7G-BD and could interfere with the analysis. HPLC fractions containing *bis*-N7G-BD and its internal standard (15.2−15.9 min) were collected, concentrated under vacuum, and reconstituted in 16 μL of buffer A for nano-LC/NSI^+^-HRMS analysis. HPLC blanks were injected after every 3 samples to monitor for any analyte carryover during offline HPLC purification. Overall, the recovery of *bis*-N7G-BD using the combined SPE and offline HPLC purification method was 78.1 ± 0.2%.

NanoLC/NSI^+^-HRMS analyses of *bis*-N7G-BD were performed using an Orbitrap QExactive mass spectrometer (Thermo-Fisher). We adopted liquid chromatography parameters outlined in our previous study [37] but utilized the high-resolution capability of the Orbitrap instrument in order to maximize the sensitivity and sensitivity of the *bis*-N7G-BD detection and quantitation. The nanoLC/NSI^+^-HRMS method was first developed and validated by processing and analyzing standard solutions in 10 μL synthetic urine containing 0.05–10.0 fmol *bis*-N7G-BD and 10.0 fmol ^15^N_6_-*bis*-N7G-BD. A linear correlation was observed between the actual and calculated amounts of *bis*-N7G-BD (*y* = 1.02*x*, *R^2^* = 0.9993) (Supplemental Figure S1). Analysis of the spiked synthetic urine samples revealed method LOD and LOQ values of 0.05 fmol and 1.0 fmol, respectively.

Control mouse urine was used to validate the nanoLC/NSI^+^-HRMS method. Standard solutions containing 0.1–10.0 fmol *bis*-N7G-BD and 10.0 fmol ^15^N_6_-*bis*-N7G-BD spiked into control mouse urine (10 μL) were processed and analyzed. A linear correlation was observed between the actual and calculated amounts of *bis*-N7G-BD in control mouse urine matrix (*y* = 1.0497*x*, *R^2^* = 0.9979; Figure 1). Based on analyses of spiked samples, the LOD and LOQ values were calculated as 0.1 fmol and 1.0 fmol, respectively (Supplemental Figure S2). Repeated analysis of *bis*-N7G-BD (1.0 fmol) spiked into control mouse urine (10 μL) (in triplicate) resulted in intra- and inter-day assay precision of 14.7% and 8.3%, respectively at the LOQ (%CV, Table 1). At 5.0 fmol analyte levels, the intra- and inter-day assay precision were 10.3% and 2.8%, respectively (%CV, Table S1). Signal suppression was observed to be 42.8 ± 2.4% comparing analyte nanoLC/NSI^+^-HRMS peak areas in the presence and absence of urine matrix. To assess stability of bis-N7G-BD in mouse urine (10 μL), a freeze–thaw stability study was performed (3.69 ± 0.27 fmol, 7.52% RSD). In summary, the method validation experiments confirmed that the new analytical method for *bis*-N7G-BD is accurate, sensitive, and specific and can be used for accurate quantification.

### 3.2. Quantitation of bis-N7G-BD in Urine of Mice Exposed to BD by Inhalation

**Figure 2:**
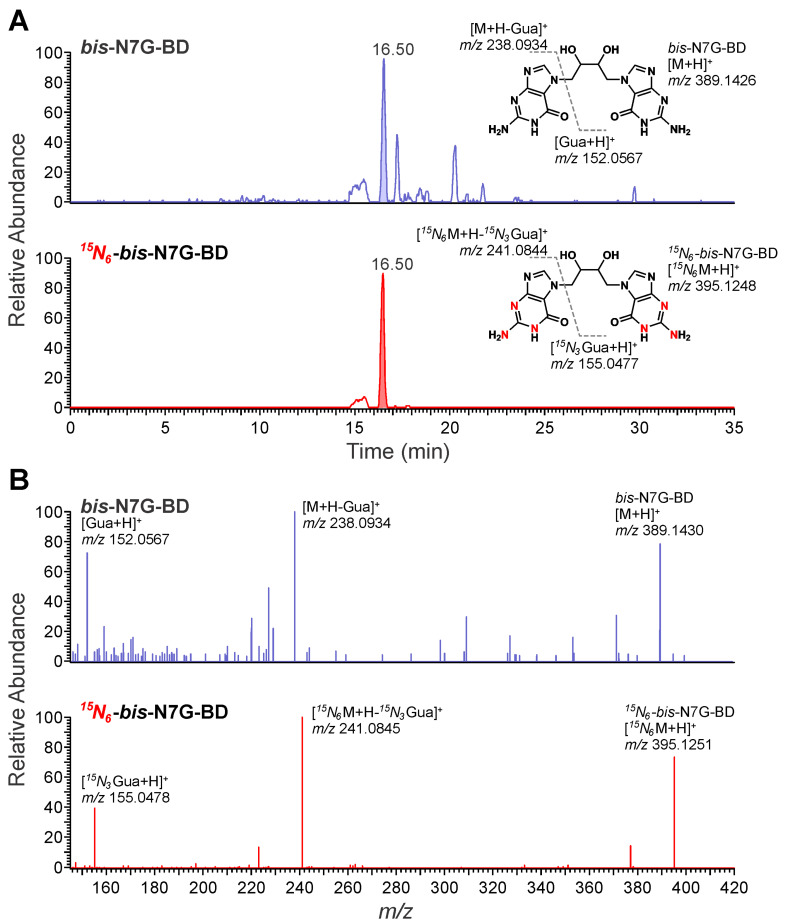
Representative extracted ion chromatogram (**A**) and MS^2^ spectra for *bis*-N7G-BD and ^15^N_6_- *bis*-N7G-BD (**B**) obtained upon nanoLC-NSI^+^-HRMS^2^ analysis of urine of laboratory mice exposed to 1,3-butadiene. Mouse urine (10 μL) was spiked with ^15^N_6_-*bis*-N7G-BD (10.0 fmol) and subjected to sample processing and nanoLC-NSI^+^-HRMS analysis on an Obitrap QExactive mass spectrometer.

**Figure 3:**
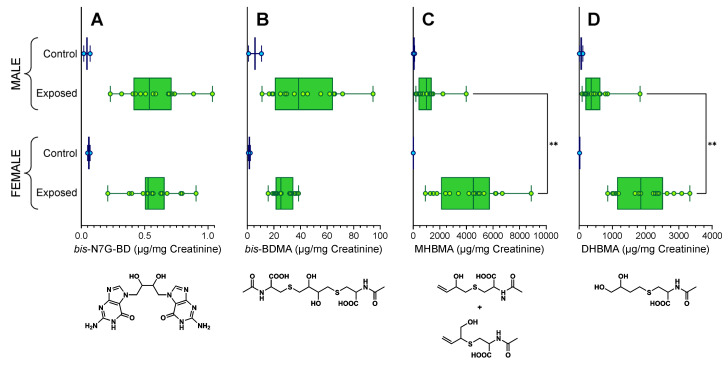
Quantitation of bis-N7G-BD DNA (**A**), bis-BDMA (**B**), MHBMA (**C**), and DHBMA (**D**) in urine of laboratory mice exposed to either filtered air (control) or 1,3-butadiene. Asterisk (**) denotes a statistically significant (*p* < 0.01) inter-sex difference (unpaired two-tailed *t*-test).

Next, the validated nanoLC/NSI^+^-HRMS method was used to quantify *bis*-N7G-BD adducts in urine of mice exposed to 590 ± 150 ppm BD by inhalation for 2 weeks (6 h/day, 5 days/week). NanoLC/NSI^+^-HRMS analysis of *bis*-N7G-BD in urine of exposed mice revealed a clear signal corresponding to *bis*-N7G-BD (Figure 2A). The nanoLC/NSI^+^-HRMS peaks exhibited excellent chromatographic peak shapes and good S/N ratios (Figure 2A). Mean levels of *bis-*N7G-BD adduct in urine of ten male mice exposed to BD was 0.574 ± 0.206 μg/mg creatinine, while mean levels of the same adduct in urine of ten female mice exposed to BD was 0.571 ± 0.163 μg/mg creatinine (Figure 3A). There was no difference in *bis*-N7G-BD excretion between male and female mice. Mean levels of *bis-*N7G-BD adduct in urine of control male mice exposed to filtered air was 0.044 ± 0.02 μg/mg creatinine (*n* = 2), while mean levels of the same adduct in urine of control female mice exposed to filtered air was 0.06 ± 0.01 μg/mg creatinine (*n* = 2, Figure 3A).

To further examine the correlation between urinary BD-derived adducts and urinary BD-mercapturic acids, the biomarkers of the toxicologically relevant and total internal dose of BD, we also quantified the urinary levels of the BD metabolites *bis*-BDMA, MHBMA, and DHBMA in the same animals (BD metabolism in Scheme 1, Supplemental Figures S3–S5). The mean levels of *bis*-BDMA, MHBMA and DHBMA metabolites in urine of the male mice exposed to BD were 43.1 ± 23.4, 1050.4 ± 954.9, 455.9 ± 433.4 μg/mg creatinine, respectively, and mean levels of the same metabolites in urine of female mice exposed to BD were 27.6 ± 7.0, 4174.1 ± 2136.9, 1978.2 ± 746.0 μg/mg creatinine, respectively (Figure 3B–D). MHBMA and DHBMA concentrations were higher in females than males for BD exposed mice (*p* < 0.01). Mean levels of MHBMA and DHBMA were 2659.5 ± 2287.2, 1240.1 ± 978.0 μg/mg creatinine, respectively (*p* < 0.01).

**Figure 4:**
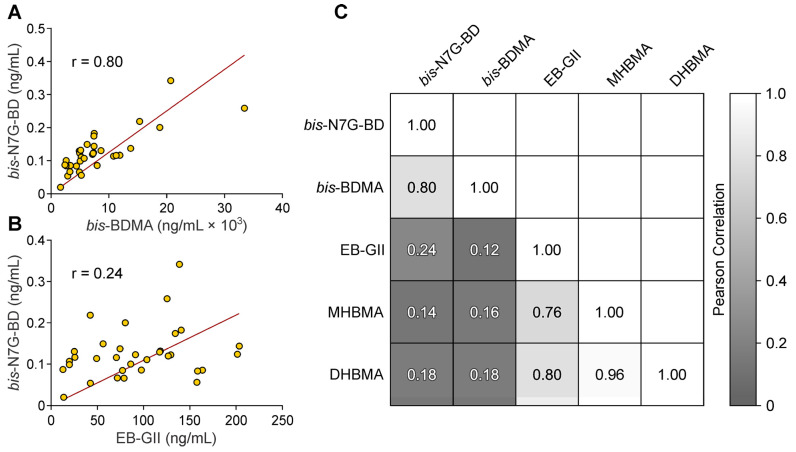
Correlation of urinary DNA adduct *bis*-N7G-BD with BD metabolites *bis*-BDMA (**A**) and EB-GII (**B**) in urine of laboratory mouse exposed to 1,3-butadiene. (**C**) Pearson correlation of urinary DNA adducts *bis*-N7G-BD and EB-GII with BD metabolites *bis*-BDMA, MHBMA, and DHBMA in urine of laboratory mouse exposed to 1,3-butadiene.

Finally, we correlated the observed levels of *bis*-N7G-BD with the BD DNA adduct EB-GII available from another study (Erber et al., submitted for publication) and BD metabolites *bis*-BDMA, MHBMA, and DHBMA measured here (Figure 4). A good correlation was observed between DEB derived DNA adduct *bis*-N7G-BD and the DEB-derived metabolite *bis*-BDMA (*r* = 0.80, Pearson correlation, *p* < 0.001). By contrast, *bis*-N7G-BD concentrations exhibited poor correlation with the EB-derived EB-GII DNA adduct and EB-derived metabolites MHBMA and DHBMA (*r* = 0.24, *p* = 0.17; *r* = 0.14, *p* = 0.45; *r* = 0.18, *p* = 0.33, respectively, Pearson correlations).

## 4. Discussion

Classified as a known human and animal carcinogen, 1,3-butadiene is metabolically bioactivated to DEB and other epoxides. DEB forms covalent DNA-DNA crosslinks including 1,4-bis-(guan-7-yl)-2,3-butanediol (*bis*-N7G-BD) [24,25,26,38]. Compared to the BD-derived mono-epoxides, DEB is nearly 200-fold more mutagenic [7]. DEB formation is thought to be responsible for a large fraction of BD-dependent genotoxic effects [23]. If left unrepaired, interstrand *bis*-N7G-BD adducts have been shown to block DNA replication and transcription, leading to genotoxicity [22,23]. Intrastrand *bis*-N7G-BD lesions are anticipated to be mutagenic [39]. As such, *bis*-N7G-BD appears to correlate with the development of cancer in laboratory animal models [25]. Because BD bioactivation to DEB is central to BD-mediated adverse health effects, quantification of DEB-derived DNA adducts following BD exposure is important. DEB-derived N7 guanine adducts such as *bis*-N7G-BD are hydrolytically unstable as a result of a positive charge on the N7 position of the alkylated base [25]. This instability results in the spontaneous release of *bis*-N7G-BD from the DNA backbone over time (t_1/2_ = 81.5 h, Scheme 1) [26]. Therefore, we hypothesized that *bis*-N7G-BD DNA adducts could be detected in urine of BD exposed laboratory animals as a biomarker of BD exposure and bioactivation to DEB.

Urinary metabolites serve as a useful, non-invasive quantitative marker of butadiene exposure and metabolism. Under physiological conditions, DNA adducts can be released from the DNA backbone via spontaneous depurination [47,48] or active repair through base excision repair pathways before excretion in the urine as a free nucleobase [49]. Other adducts are excreted in a nucleotide or nucleoside form following repair by nucleotide excision repair mechanism [50,51,52] and/or nucleolytic degradation of DNA [53,54]. Earlier studies have described urinary DNA adducts induced by endogenous biomolecules such as estrogens [55], lipid peroxidation products [56] as well as by reactive exogenous metabolites such as styrene [57] and polyaromatic hydrocarbons [58]. Previous studies employed BD-derived urinary adduct, EB-GII, as a useful biomarker of exposure to BD and metabolic activation [34,35,36,59]. However, EB-GII is formed from BD monoepoxide (EB) and thus does not reflect the formation of the most genotoxic metabolite of 1,3-butadiene (i.e., DEB).

In this study, we developed a quantitative method for DEB-derived *bis*-N7G-BD adducts in urine. Unlike tissue or blood samples, urine can be collected in a non-invasive manner, is easily stored, and is readily available from many epidemiological studies. Highly advanced sample enrichment procedures were adopted to isolate urinary *bis*-N7G-BD adducts as urine is a highly complex biological matrix. These enrichment procedures were coupled with highly sensitive and specific nanoLC/NSI^+^-HRMS analysis. This analytical method is comparable in sensitivity to a previously reported nanoLC/NSI^+^-MS/MS method for *bis*-N7G-BD from tissue DNA (LOQ, 1.0 fmol) [37]. *Bis*-N7G-BD adducts were quantified in urine of male and female mice exposed to high concentrations of BD (590 ± 150 ppm BD for 2 weeks) via inhalation. MS^2^ spectra of urinary *bis*-N7G-BD adducts detected in urine samples matched the spectrum of authentic *bis*-N7G-BD standard (Figure 2A). Isotope dilution with ^15^N-labeled standard allowed for accurate quantitation of *bis*-N7G-BD.

*Bis*-N7G-BD adducts have been previously identified and quantified in genomic DNA of laboratory mice exposed to both ambient BD concentrations (0.3 to 3 ppm) [37] and relatively high BD concentrations (6.25–625 ppm) [25]. At high BD concentrations (>62.5 ppm), metabolic activation of EB to DEB in mice is saturated [25]. Liver *bis*-N7G-BD [25] and EB-GII DNA adduct levels [60] have been quantified in B6C3F1 mice exposed to 625 ppm BD for 2 weeks (6 h/d, 5 d/wk). These studies revealed 3.95 *bis*-N7G-BD DNA adducts per 10^7^ nucleotides and 2.4 EB-GII adducts per 10^6^ representing a 6.07-fold difference between the two adducts. By contrast, when comparing the urinary EB-GII levels in these mice (508.238 μg/mg creatinine) (Erber et al., submitted for publication) to the levels of *bis*-N7G-BD (0.572 μg/mg creatinine), there was nearly 890-fold difference (see above). The significantly lower levels of *bis*-N7G-BD as compared to EB-GII in mouse urine may be due to a greater efficiency in release of BD monoadducts from the DNA backbone, potential excretion of *bis*-N7G-BD in bile [61], or further metabolism of *bis*-N7G-BD adduct. We examined the possibility that *bis*-N7G-BD are metabolized through oxidation, hydrolysis or deamidation. Oxidative metabolism of urinary adducts has been previously observed for malondialdehyde lesions [62]. However, targeted analysis using nanoLC/NSI^+^-HRMS did not reveal any signals corresponding to proposed *bis*-N7G-BD metabolic products (Supplemental Figure S6).

Availability of specific urinary biomarkers for EB and DEB (*bis*-BDMA, MHBMA and DHBMA) permits evaluation of formation of BD-derived electrophilic metabolites. *Bis*-BDMA, MHBMA and DHBMA were observed at levels higher than previously reported. When rats were exposed to 200 ppm BD by inhalation for a similar time duration (6h/d, 5 d/wk, 2 wk), *bis*-BDMA, MHBMA, and DHBMA were reported as 4.8 ± 2.9, 77.9 ± 37.8, and 209.5 ± 85.1 μg/mL urine, respectively [17]. In our study, mice were exposed to 590 ppm BD by inhalation for the same duration, and *bis*-BDMA, MHBMA, and DHBMA were observed as 8.09 ± 6.3, 593.4 ± 532.7, and 280.1 ± 235.4 μg/mL urine, respectively. These higher levels can be explained by the increased BD exposure concentrations and more efficient bioactivation of BD to DEB in the mouse as compared to rats or humans [16,25].

In agreement with their formation from the same BD metabolite, DEB-derived *bis*-BDMA and *bis*-N7G-BD exhibited a good correlation (Figure 4A). Significant correlation was also observed among EB-derived species MHBMA, DHBMA and EB-GII (Figure 4C). In contrast, correlation between DEB-derived bis-N7G-BD and EB-derived EB-GII was poor (Figure 4B). Potential correlation between urinary *bis*-BDMA and *bis*-N7G-BD has not been previously examined. However, an association study in rat comparing urinary *bis*-BDMA and *bis*-N7G-BD in liver DNA exhibited poor correlation between the two biomarkers at high BD concentrations (>62.5 ppm) [17]. This may be explained by possible interspecies differences, and exposure reaching saturation levels of *bis*-BDMA in mice.

In summary, this study establishes *bis*-N7G-BD as a novel urinary biomarker of exposure to BD and metabolic bioactivation of BD to DEB. To accomplish this aim, we developed and validated a quantitative isotope dilution nanoLC/NSI^+^-HRMS method for urinary *bis*-N7G-BD adducts. This method exhibits excellent selectively, accuracy, and sensitivity. The methodology developed in this study will be useful for future studies of BD exposure and metabolism.

## Figures and Tables

**Table 1:** Method validation results for nanoLC-NSI^+^-HRMS analysis of *bis*-N7G-BD (1 fmol) spiked into control mouse urine (10 μL).

Day	Mean	RSD (%)	Accuracy (%)	*N*
1	1.01 ± 0.12	14.7	101.8 ± 15.0	3
2	1.16 ± 0.09	10.0	116.9 ± 11.7	3
3	1.19 ± 0.07	7.8	119.2 ± 5.1	3
Interday	1.12 ± 0.07	8.3	112.6 ± 7.7	9

## Supplementary Materials

The following are available online at https://www.mdpi.com/article/10.3390/toxics9100247/s1, Figure S1: NanoLC/NSI^+^-HRMS method validation: correlation between spiked and the observed amounts of *bis*-B7G-BD spiked into 10μL water, Figure S2: NanoLC/NSI^+^-HRMS analysis of *bis*-N7G-BD and [^15^N_6_]-*bis*-N7G-BD spiked in unexposed mouse urine, Figure S3: Bis-BDMA nanoLC/ESI^−^—MS/MS method validation, Figure S4: MHBMA HPLC/ESI^−^-HRMS method validation, Figure S5: DHBMA HPLC/ESI^−^-HRMS method validation, Figure S6: NanoLC/NSI^+^-HRMS identification of metabolic products of *bis*-N7G-BD in BD-expose mouse urine, Table S1: Method validation results for nanoLC-NSI^+^-HRMS analysis of bis-N7G-BD (5 fmol) spiked into control mouse urine (10 μL).

## Abbreviations

BD, 1,3-butadiene; DEB, 1,2,3,4-diepoxybutane; *bis*-N7G-BD, 1,4-*bis*-(gua-7-yl)-2,3-butanediol; EB-GII, N7-(1-hydroxy-3-buten-2-yl) guanine; *bis*-BDMA, 1,4-*bis*-(N-acetyl-L-cystein-S-yl)butane-2,3-diol; MHBMA, 2-(N-acetyl-L-cystein-S-yl)-1-hydroxybut-3-ene/1-(N-acetyl-L-cystein-S-yl)-2-hydroxybut-3-ene; DHBMA 4-(N-acetyl-L-cystein-S-yl)-1,2-dihydroxybutane; CID, collision-induced dissociation; nano-LC/ESI^−^-MS/MS, isotope dilution nanoLC electrospray tandem mass spectrometry; nano-LC/NSI^+^-HRMS, isotope dilution nanoLC nanospray high resolution mass spectrometry; SPE, solid phase extraction, SRM, selected reaction monitoring mode; PRM, parallel reaction monitoring mode
