# Supplementary material for: Quantitative NanoLC/NSI+-HRMS Method for 1,3-Butadiene Induced bis-N7-guanine DNA-DNA Cross-Links in Urine
Source: Toxics. 2021 Oct 2;9(10):247. doi: 10.3390/toxics9100247 (PMC8540193; doi:10.3390/toxics9100247)
Supplement: Supplementary file 1 [file toxics-09-00247-s001.zip › toxics-1367891-supplementary.pdf]

toxics

MDPI

Supplementary Materials: Quantitative NanoLC/NSI+ -HRMS Method for 1,3-Butadiene Induced bis-N7-guanine DNA-DNA Cross-links in Urine

Luke Erber, Samantha Goodman, Caitlin C. Jokipii Krueger, Ivan Rusyn and Natalia Tretyakova

Table S1. Method validation results for nanoLC-NSI+ HRMS analysis of bis-N7G-BD (5 fmol) spiked into control mouse urine (10 μL).

Day	Mean	RSD (%)	accuracy (%)	*N*
1	5.36 ± 0.06	1.4	107.3 ± 1.5	3
2	5.32 ± 0.34	7.9	106.5 ± 8.4	3
3	5.09 ± 0.42	10.3	101.8 ± 10.4	3
Interday	5.26 ± 0.12	2.8	105.2 ± 2.4	9

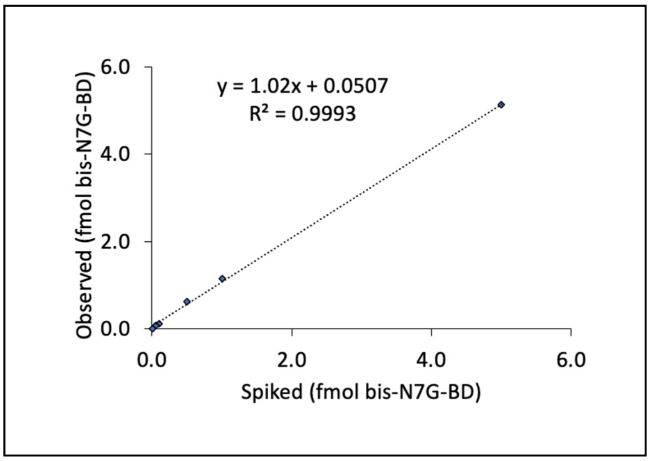

Figure S1. NanoLC/NSI+-HRMS method validation: correlation between spiked and the observed amounts of bis-B7G-BD spiked into 10μL synthetic urine. Spiked amounts were 0, 0.01, 0.5, 1, 2, or 5 fmol of bis-N7G-BD and 5 fmol of $^{15}N_6$- bis-N7G-BD (internal standard), followed by sample processing and nanoLC/ESI+-HRMS analysis.

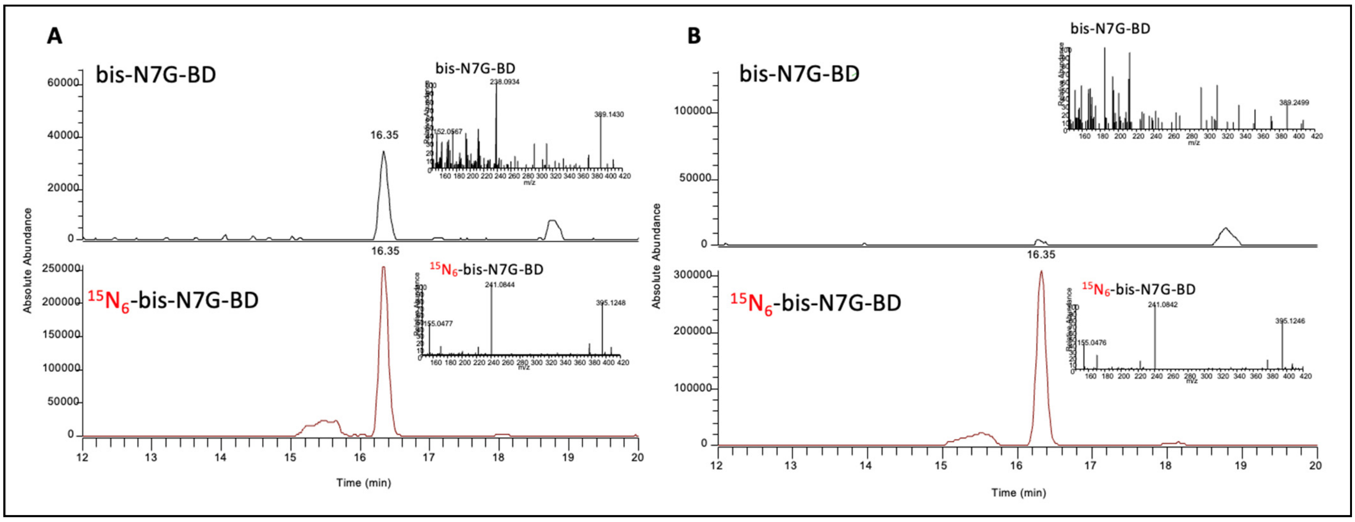

Figure S2. NanoLC/NSI+ HRMS analysis of unexposed mouse urine (10 μL), spiked with bis-N7G-BD (1 fmol, LOQ sample) and [$^{15}N_6$]-bis-N7G-BD (10 fmol) (**A**) and unexposed mouse urine sample with endogenous levels of bis-N7G-BD, spiked only with [$^{15}N_6$]-bis-N7G-BD (10 fmol) (**B**). Results are shown with a 5 ppm mass tolerance.

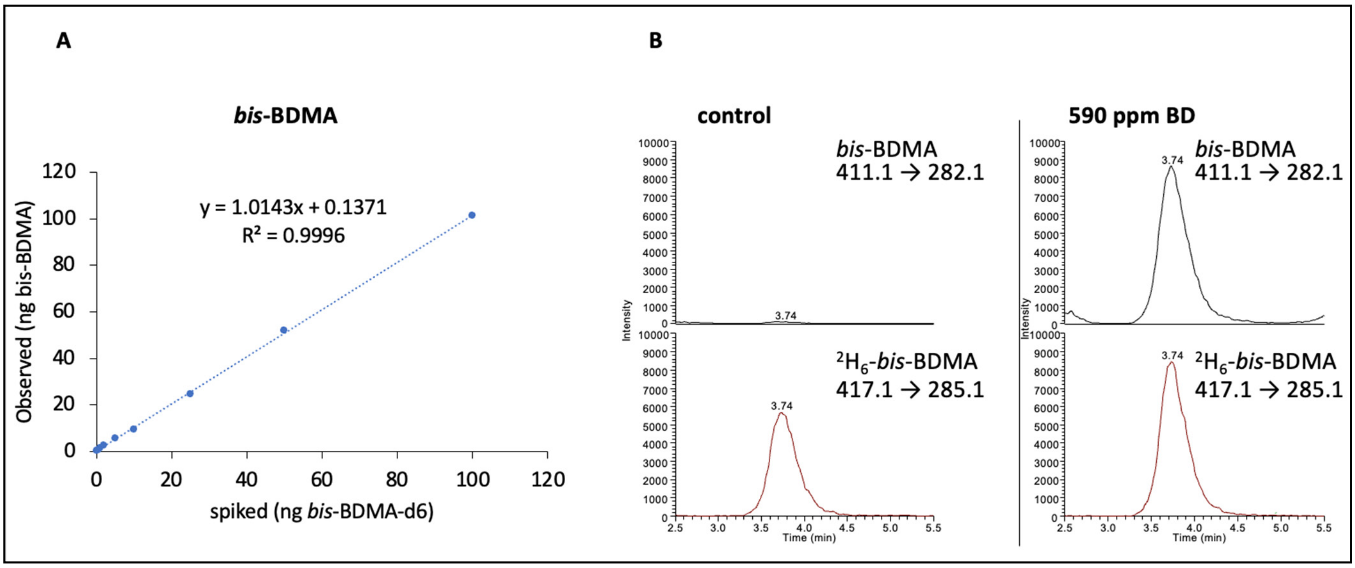

Figure S3. Bis-BDMA nanoLC/ESI$^-$-HRMS method validation. (**A**) NanoLC/ESI$^-$-MS/MS method validation: correlation between the spiked and the observed amounts of *bis*-BDMA spiked into control mouse urine (10 μL). (**B**) Representative extracted ion chromatogram for *bis*-BDMA in urine of 1,3-butadiene-exposed and control mice (10μL).

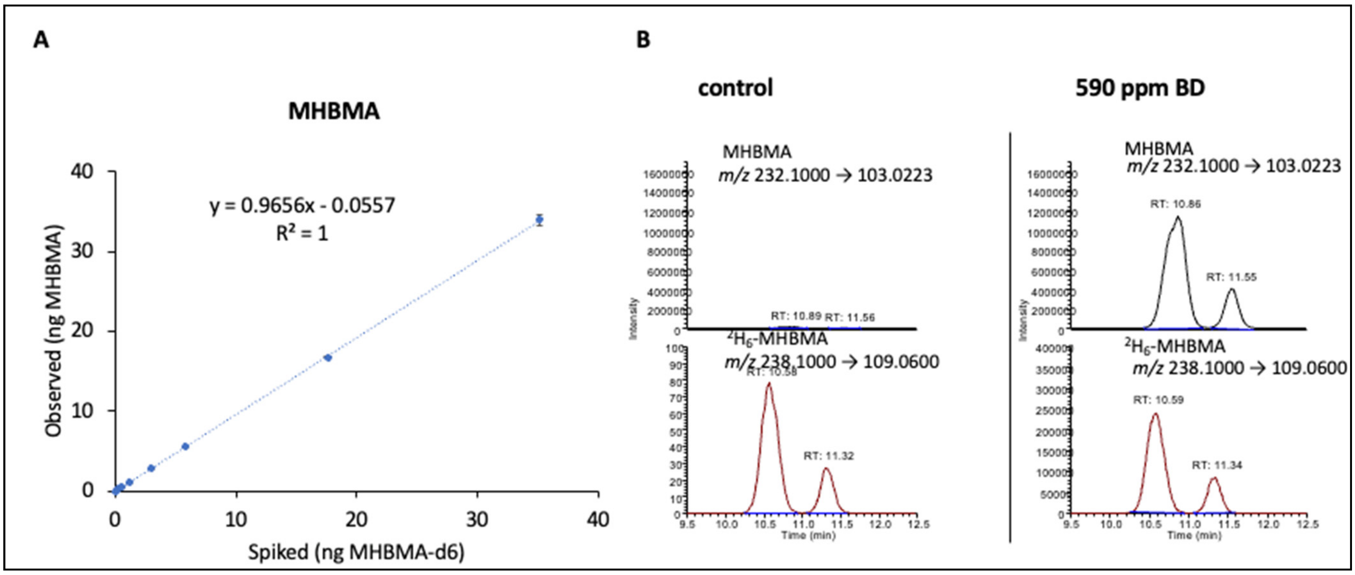

Figure S4. MHBMA HPLC/ESI$^-$-HRMS method validation. (**A**) NanoLC/ESI$^+$-HRMS method validation: correlation between the spiked and the observed amounts of MHBMA spiked into control mouse urine (20 μL). (**B**) Representative extracted ion chromatogram for MHBMA in urine of 1,3-butadiene-exposed and control mice (20 μL).

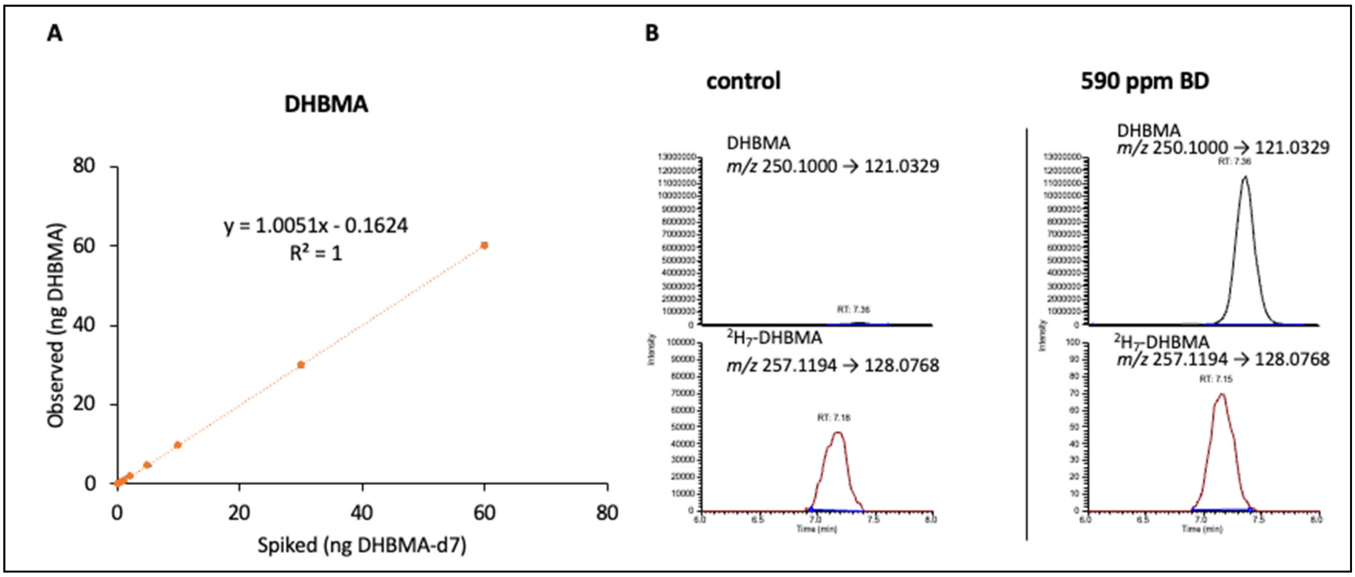

Figure S5. DHBMA HPLC/ESI^--HRMS method validation. (**A**) Correlation between the spiked and the observed amounts of DHBMA spiked into control mouse urine (20 µL). (**B**) Representative extracted ion chromatogram for DHBMA in urine of 1,3-butadiene-exposed and control mice (20 µL).

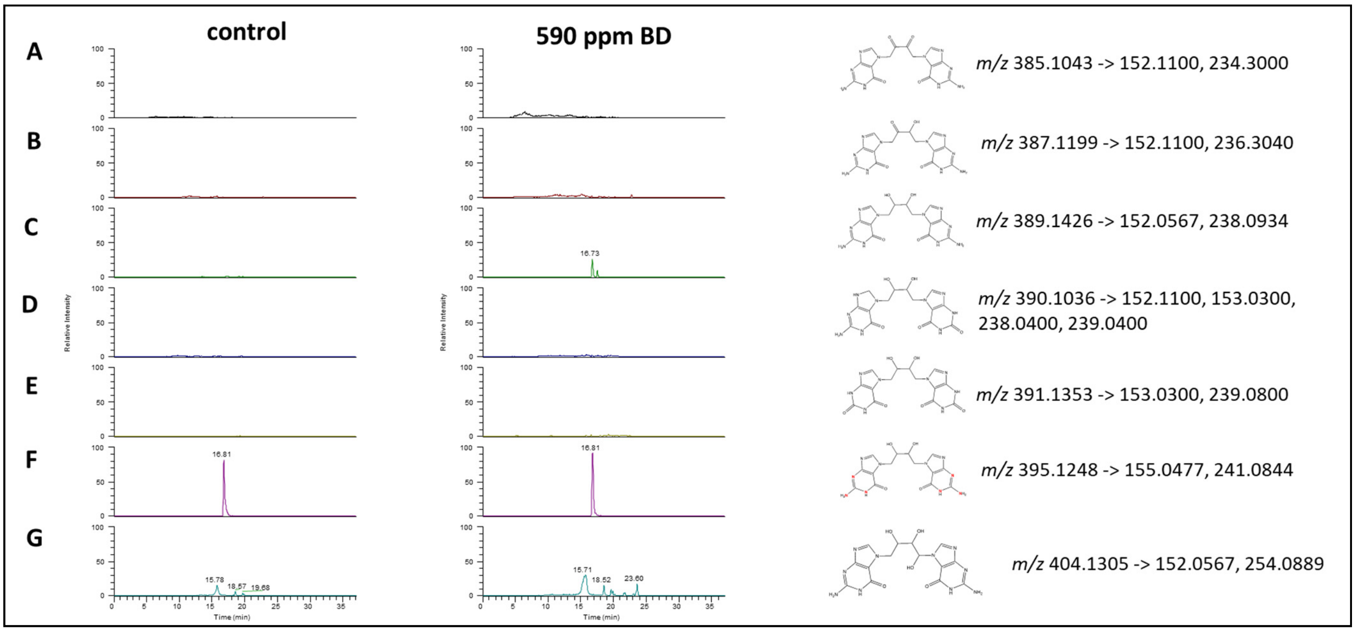

Figure S6. NanoLC/NSI+ HRMS identification of metabolic products of bis-N7G-BD in BD-expose mouse urine. (**A**) bis-N7G-BD oxidized twice, (**B**) bis-N7G-BD oxidized once, (**C**) bis-N7G-BD, (**D**) bis-N7G-BD deamidated once, (**E**) bis-N7G-BD deamidated twice, (**F**) $[^{15}N_6]$-bis-N7G-ND and (G) bis-N7G-BD containing three hydroxyl groups on the guanine guanine butadiene-derived alkyl linker.
